# Terson’s Syndrome – case report

**DOI:** 10.22336/rjo.2017.8

**Published:** 2017

**Authors:** Andreea Moraru, Ruxandra Mihailovici, Dănuţ Costin, Daniel Brănişteanu

**Affiliations:** * “Grigore T. Popa” University of Medicine and Pharmacy, Iaşi, Romania; ** “Prof. N. Oblu” Hospital, Iaşi, Romania

**Keywords:** Terson’s syndrome, persistent hemorrhage, epiretinal membrane, vitrectomy

## Abstract

Terson’s Syndrome is represented by a vitreous, retrohyaloid, retinal, or subretinal hemorrhage occurring consequent to an acute intracranial hemorrhage or elevated intracranial pressure. The outcome may include a complete clearing of the blood and the restoration of VA or persistent hemorrhage.

This report presents the case of a 43-year-old woman who underwent bilateral surgery for a persistent vitreous hemorrhage and a hematoma underneath the internal limiting membrane in the left eye. The event followed shortly after a subarachnoid hemorrhage due to the rupture of a posterior communicating artery aneurism. Vitrectomy was performed in both eyes, together with the peeling of the internal limiting membrane in the left eye, followed by a bilateral good outcome.

## Introduction

Terson’s syndrome is represented by a vitreous, retrohyaloid, retinal, or subretinal hemorrhage occurring consequent to an acute intracranial hemorrhage or elevated intracranial pressure.

It was first described by the French ophthalmologist Albert Terson in the beginning of the 1900’s [**1**]. Terson’s syndrome has been most commonly described in subarachnoid hemorrhages due to cerebral aneurisms, head trauma, intracranial elevated pressure, and tumors and intracranial hemorrhage, which occurs during or post operatively. 

The pathogenesis of Terson’s Syndrome has been controversial, but there are 2 main accepted mechanisms [**2**,**3**]. One of them states that elevated intracranial pressure has a crucial role, causing the raise of intraocular venous pressure and the rupture of the superficial vessels, hence the hemorrhage. The other one asserts that the accumulated blood form the subarachnoid space enters the eye along the optic nerve and retinal vessels space, producing a vitreous or retrohyaloid hemorrhage. 

## Case report

A 43-year-old woman referred to the Ophthalmology Clinic complaining of a sudden decrease in visual acuity, which occurred after she had surgery for a ruptured intracranial aneurism.

The patient was admitted to the Neurosurgery Department, a few weeks prior for loss of consciousness. She was diagnosed with rupture of a posterior communicating artery aneurism, which was clipped along with its origin from the internal carotid artery. Two weeks postoperatively, the patient accused a sudden decrease of visual acuity in both eyes and she was directed towards the Ophthalmology Clinic.

Upon examination, the patient presented with BCVA RE = 0,3 and BCVA LE = 0,02, normal IOP and no significant changes in the anterior pole.

**Fig. 1:**
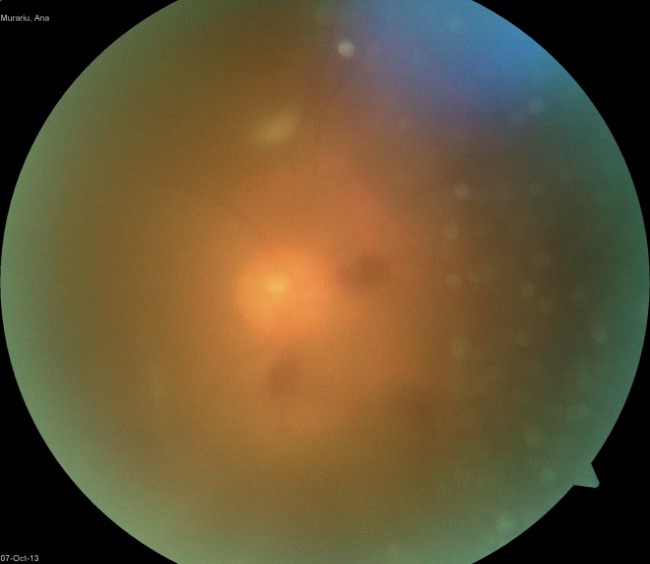
RE Fundus: diffuse hemorrhage

**Fig. 2:**
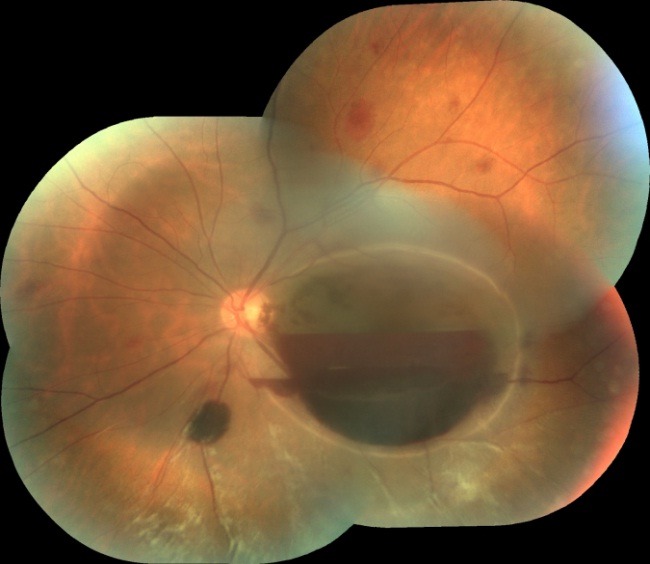
LE Fundus: double hemorrhagic level and hematoma in the macular area

Fundoscopic examination revealed diffuse vitreous hemorrhage in both eyes, denser in the right eye (**Fig. 1**). Also, the left eye had a double hemorrhagic level: retrohyaloid hemorrhage in the macular area and hematoma under the internal limiting membrane (**Fig. 2**).

**Fig. 3:**
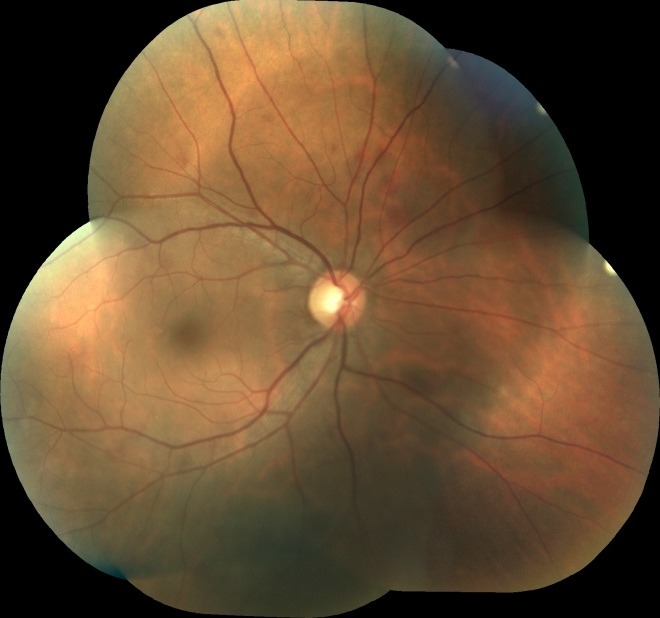
RE Fundus: normal aspect

Taking into account the fact that both eyes have been affected by vitreous hemorrhage in this young patient for over three weeks, we decided to perform vitrectomy. 23G Pars plana posterior vitrectomy was performed in the right eye. Three weeks postoperatively, the fundoscopic aspect of the right eye was normal (**Fig. 3**).

Due to the persistence of the hemorrhage, vitrectomy was also performed in the left eye. Tissue plasminogen activator was injected intravitreal one day before surgery, to breakdown the blood clot (25 µg in 0,05 mL). Subsequently, 23G pars plana posterior vitrectomy and peeling of the internal limiting membrane was performed.

**Fig. 4:**
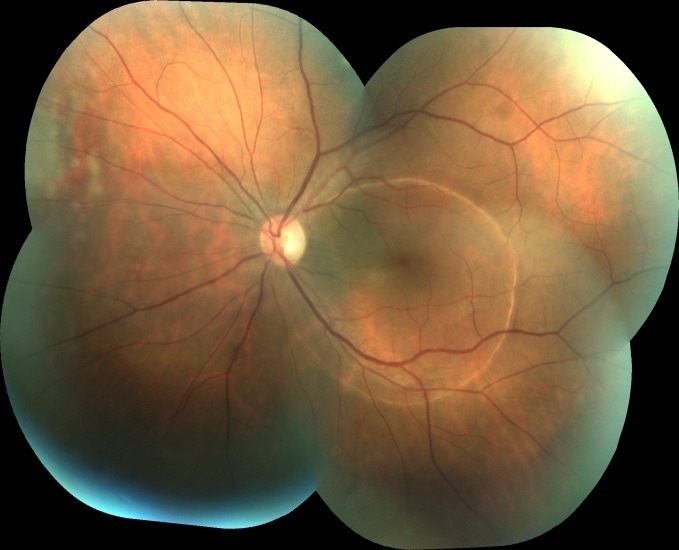
LE Fundus: normal aspect

Postoperative outcome was favorable with a normal aspect of the retina and the entire fundus (**Fig. 4**).

At 1, 3 and 6 months follow-ups the BCVA of the RE was 1 and the BCVA of the LE was 0,8 and the fundus aspect of both eyes was stationary.

A few pathological entities could be discussed for the purpose of differential diagnosis. Regarding the vitreous hemorrhage, the differential diagnosis could be made with:

1. Advanced stage diabetic retinopathy: the patient had no prior history of diabetes;

2. Trauma: there was no history of trauma;

3. Hemoglobinopathies: the blood count was within normal limits.

The differential diagnosis of the sub- internal limiting membrane hematoma comprised:

1. Purtscher’s retinopathy: it has a varied etiology, such as severe head trauma, acute pancreatitis, lupus, chronic renal disease. The mechanism of this disease consists of intravascular micro particles occluding the arterioles (fibrin clots, platelet – leukocyte aggregates, fat emboli, gaseous emboli). Its manifestations consist of intraretinal hemorrhages and cotton-wool exudates surrounding the optic nerve head and later on, the optic nerve atrophies [**4**];

2. Valsalva retinopathy: it is secondary to a sudden increase in intrathoracic or intraabdominal pressure (weight lifting, coughing, sneezing, vomiting). This mechanism causes an elevation in the intraocular venous pressure and further on, a spontaneous rupture of the retinal capillaries. Ocular findings are classically described as uni/ bilateral hemorrhages in the macular area, underneath the internal limiting membrane, retinal/ subretinal/ vitreous hemorrhages, which evolve towards spontaneous clearing of the blood without long-term complications.

3. Hypertensive retinopathy: it represents the ophthalmic findings secondary to systemic arterial hypertension, which, in an acute, significant rise of pressure may cause the constriction of arterial vascular beds and the obstruction of retinal arterioles. These results in cotton-wool spots near the optic nerve head, nerve fiber layer hemorrhages in the peripapillary region, lipid exudates in the macula, macular edema, and retinal hemorrhages [**4**].

## Discussions

Terson syndrome is usually described in correlation with ruptured cerebral vessel aneurysms, mainly in three locations: in the internal carotid artery, the middle cerebral artery bifurcation, and in the upper part of the basal artery [**5**].

The sudden elevation of the intracranial pressure has a crucial role in Terson’s syndrome. It causes the raise of intraocular venous pressure and the rupture of the superficial vessels, hence the hemorrhage. Also, the pressure is transmitted along the optic nerve sheath and retinal vessels space, occluding the retinal and choroidal anastomoses at the lamina cribrosa.

Approximately 20% of the patients diagnosed with subarachnoid hemorrhage present with Terson’s syndrome. This association has a negative influence on the mortality rate. Patients diagnosed with Terson syndrome have a 40-60% mortality rate, 3 to 9 times higher comparative to the patients who only present with subarachnoid hemorrhage unaccompanied by ocular manifestations [**6**].

Most often, the patient is neurologically impaired and the visual acuity cannot be tested, but the degree of vision loss is usually related to the extent of the intraocular hemorrhage. It can range from 20/ 20 to light perception. Also, the amount of intraocular hemorrhage is influenced by the speed of accumulation and magnitude of the intracranial pressure elevation [**7**]. 

Usually, the intraocular hemorrhage is bilateral and superficial and infrequently intraretinal or subretinal. A preretinal hemorrhage can cause a vitreous hemorrhage weeks after the initial event. 

According to literature data, the incidence of intraocular hemorrhage associated with subarachnoid hemorrhage is 10-50% [**8**]. Vitreous hemorrhage incidence is lower: 3-13% [**9**].

The histopathological specimens obtained from patients with Terson’s syndrome have displayed the presence of erythrocytes and leukocytes in the vitreous, subhyaloidal, and subinternal limiting membrane space and in the retina. Although not as common, some studies report the presence of subretinal blood. Also, the examinations of epiretinal membranes showed glial cells and basement membrane material [**10**].

The formation of an epiretinal membrane is one of the most common complications which can occur in Terson’s, with an incidence of up to 78%. It is a result of the fibroblast and glial cell proliferation, which can occur in the subhyaloidal or subinternal limiting membrane space created by the intraocular hemorrhage [**10**]. It can critically affect the patients’ vision after the clearing of the hemorrhage and it can become significant even 4 years after the hemorrhagic episode. Other reported long-term complications include retinal pigment epithelium mottling, optic atrophy, macular holes, retinal folds, cystoid retinal changes, proliferative vitreoretinopathy, retinal detachment, and cataract formation [**11**].

Glatt și Machemer have demonstrated that blood has a toxic effect over the retina’s photoreceptors, especially in the first 7 days after the hemorrhage [**12**]. The iron from the hemoglobin catalyses the conversion of hydrogen peroxide into hydroxyl radical, which is the most destructive species of reactive oxygen. The destruction caused by this radical consists of the peroxidation of lipids, breaking of DNA chains and biomolecular degradation. Since the main function of the retinal pigment epithelium (RPE) is to phagocyte the photoreceptors external segments, which are rich in lipids, the retina, and the RPE are prone to oxidative damage [**13**].

The visual prognosis of the patients who survive a subarachnoid hemorrhage is favorable. Most of the vitreous hemorrhages spontaneously clear up [**14**]. Only 40% of the cases need a vitrectomy and only half of these also need a peeling of the internal limiting membrane [**15**]. Vitrectomy is indicated in the cases showing persistent or bilateral vitreous or macular hemorrhages. Recent studies suggest that an early vitrectomy may help with a fast restoration of vision, thus reducing the incidence of complications that can occur, such as proliferative vitreoretinopathy and glaucoma [**16**].

Kuhn et al. have described the accumulation of blood underneath the internal limiting membrane in Terson’s syndrome and reported a 39% incidence of macular hemorrhages [**17**]. Proceeding with a vitrectomy has lead to good results in all these cases, over 80% of the patients having a final VA of 0,8 or more. The best results were achieved in young patients (under 45 years) and in those who were operated on during the first 3 months [**16**].

In 1991, Lewis has introduced the tissue plasminogen activator (tPA) in the treatment plan, helping with the breaking down of the blood clot in cases of submacular hemorrhages [**18**]. The tPA is a protease that transforms plasminogen in plasmine which, subsequently, breaks the fibrin clot. It can be used as a subretinal injection during the vitrectomy or it can be injected intravitreously along with the pneumatic displacement of the clot [**19**]. It has been demonstrated that intravitreously administered tPA is toxic in doses over 100 µg [**20**].

Taking into account the young age of our patient and the fact that the vitreous hemorrhage was persistent and bilateral, with macular involvement in the left eye, we decided that vitrectomy was necessary in this case in order to prevent the occurrence of further complications and to improve the quality of life. The results achieved in this case were comparable to those described in the specialty literature. Both eyes regained a good visual acuity immediately after surgery. The final VA of the left eye was lower than the VA of the other eye (0,8 comparative to 1), suggesting that the persistence of blood in the macular area influenced the functional prognosis. 

The final functional prognosis is influenced by many factors: the age of the patient, the rate of pre and postoperative complications such as epiretinal membranes and cataract formation. Some authors suggest that the final visual acuity influences the neurological condition of the patient, regarding the damage to brain structures associated with Terson syndrome [**21**,**22**].

## Conclusions

Vitrectomy is a safe and efficient procedure of treating intraocular hemorrhage, which is secondary to a ruptured intracranial aneurism, and has also enabled a quick recovery of visual acuity in this young patient.
